# Young women’s food consumption and mental health: the role of employment

**DOI:** 10.1186/s12905-022-01675-4

**Published:** 2022-03-25

**Authors:** Jaewon Lee, Jennifer Allen

**Affiliations:** 1 School of Social Welfare, Inha University, 100 Inha-ro, Michuhol-gu, Incheon, 22212 Republic of Korea; 2 School of Social Work, Michigan State University, East Lansing, MI USA

**Keywords:** Young women, Mental health, Employment, Healthy, Unhealthy foods

## Abstract

**Objectives:**

This study explores the relationship between young women’s consumption of healthy and unhealthy food and depression and examines the moderating effect of their employment status on the relationship.

**Methods:**

The National Longitudinal Survey of Youth 79 for Children and Young Adults (NLSY79 CY) was used for this study. The final sample included a total of 1524 young women aged from 18 to 35 years. Multiple Linear Regression was conducted to answer the research questions.

**Results:**

Fast food consumption was related to higher levels of depression among young women while fruit intake was associated with lower levels of depression. Employment status moderated the relationship between young women’s fruit consumption and depression.

**Conclusions:**

Young women are encouraged to consume more fruit and less fast food in order to ameliorate depression. Programs that target young women’s mental health should consider addressing their nutritional needs, through, for example, vouchers for fresh, nutritious foods; nutrition or cooking education; or having a certified nutrition specialist on staff.

## Introduction

Research has shown that young adults consume high amounts of fast food and soda or sugar-sweetened beverages and low amounts of fruits and vegetables [1–6]. For the purpose of this study, we define soda or sugar-sweetened beverages and fast food as unhealthy foods, and fruits and vegetables as healthy foods. Moreover, compared to other age groups, young adults are more likely to exhibit symptoms of depression, and young women are more likely to report depression than young men [7–14]. Further, researchers have found that fast food and soda consumption are positively associated with depression among young women, while fruit and vegetable consumption are negatively associated with depression [15–18]. Scientists have found that nutrition and food consumption affects mood and depression through a variety of nutritional deficiencies, which thereby affect neurotransmitters in the brain that are associated with depression, including serotonin, dopamine, and noradrenaline [19]. Therefore, the relationship between food consumption and depression is of interest for physical and social scientists alike.

In this study, we also examine unemployment, which is highest in young adulthood [20–22]. Researchers have also found that unemployment in young adulthood is positively associated with depression [23–25], as well as an increased consumption of fast food and soda and decreased consumption of fruits and vegetables [26]. Therefore, studies have shown that food consumption is associated with unemployment and depression, and also that unemployment is associated with depression. However, little is known about the moderating role of unemployment on the relationship between food consumption and depression. In this study, we address this gap in the literature by examining the relationship between food consumption and depression with unemployment as a moderator, particularly among young women. To explore these relationships, we utilized cross-sectional data from one wave of the National Longitudinal Survey of Youth 79 for Children and Young Adults (NLSY79 CY) collected in 2014.

### Young women’s food consumption

Researchers have found differences in the food consumption of young men and women [5, 27–33]. From adolescence to older adulthood, males are more likely than females to drink soda and consume fast food [5, 28–33]. There is also a gender difference in the pattern of fast food consumption, with men more likely than women to consume fast food for lunch (48.3% vs. 39.1%), while women are more likely to consume fast food as a snack (25.7% vs. 19.5%) [27]. However, females are more likely than males to consume fruits and vegetables [34, 35].

Despite these gender differences across multiple age groups in healthy and unhealthy food consumption, it is of interest to examine young women’s food consumption. Researchers have found that women are more likely to consume soda or other sugar-sweetened beverages and fast food and less likely to consume fruits and vegetables during young adulthood than in later periods of adulthood [1–6, 32, 33, 36, 37]. Compared to adolescents and children, young adults’ consumption of healthy and unhealthy foods are relatively similar [38], with some evidence suggesting that children and adolescents consume slightly more fast food and soda than young adults [2, 39].

### Prevalence of depression among young women

The prevalence of major depression in young adulthood is high, and it is most likely to emerge during this developmental period [8, 10, 11]. Results from the 2017 National Survey of Drug Use and Health reveal that the prevalence of major depression was highest during young adulthood (ages 18 to 25), at 13.1% [10]. Results from the National Epidemiologic Survey on Alcohol and Related Conditions-III similarly revealed that young adults were more likely to be diagnosed with major depression than older adults [8]. Moreover, researchers have found that the prevalence of major depression in young adulthood is increasing over time [40, 41]. From 2005 to 2014, young adults’ prevalence of major depression increased from 8.8 to 9.6% [41]. Additionally, from 1998 to 2017, the percentage of young adults reporting at least two symptoms of major depression in the past 30 days increased among those aged 18 to 24 years (4.4–7.3%), as well as among those aged 25 to 29 years (4.6–5.5%) [40].

Researchers also consistently have found that females are more likely to experience major depression than males across most ages [7–9, 12, 42]. These findings were consistent across cultures and in a meta-analysis of 90 studies [7, 9]. Researchers have found that before puberty, girls and boys are equally likely to exhibit symptoms of depression, but this prevalence peaks at ages 14 to 25 and decreases with age, although women remain more likely to report depression than men [7]. In one sample of college athletes, female athletes were 1.84 times more likely to experience major depression than their male counterparts [14]. Additionally, researchers found that female college students are more likely than male college students to experience depression overall and to report symptoms of physiological agitation related to depression [43].

### Food consumption and depression

#### Unhealthy food consumption and depression

Four studies examined the relationship between fast food or soda consumption and depression in young adulthood [15, 16, 18, 44]. Findings on the relationship between fast food consumption and depression have been mixed, suggesting more research is needed in this area. One of these studies found a positive association between fast food consumption and depression in male and female college students in the United Kingdom [15]. In another, fast food consumption was positively associated with depression only among Mexican female college students, but not their male counterparts [16]. In the third, there was no significant relationship found between fast food consumption and depression in a sample of Lebanese college students [44]. Moreover, findings on the relationship between soda consumption and depression in young adulthood are similarly mixed [15, 18]. In a sample of Chinese college students, those students who drank soda more than seven times weekly were more depressed than students who infrequently drank soda [18]. However, in another sample of college students in the United Kingdom, soda consumption was not significantly associated with depression [15]. Additionally, although not focused on young adulthood specifically, results from a meta-analysis revealed a positive association between depression and soda consumption that did not vary by gender, country, alcohol consumption, smoking or level of physical activity [45]. Further, researchers examined gender differences in the relationship between sugar-sweetened beverage consumption and depression, and in two studies the relationship was stronger among women than men [46, 47].

#### Healthy food consumption and depression

Two studies examined the relationship between vegetable and fruit consumption and depression in young adulthood. These studies both revealed that the consumption of more fruits and vegetables was associated with lower levels of depression among college students in multiple countries [15, 17]. Further, these relationships may differ by gender; researchers found significant associations between increased vegetable consumption and fruit consumption and lower depression among adult women but not among adult men [48].

### The role of employment on the relationship between young women’s food consumption and depression

Although young adult employment rates in the United States have been steadily decreasing from a high of 18.29% in 2010, unemployment rates in October 2018 were still highest among those aged 16 to 24 (8.3%) and lowest among those aged 45 to 54 (2.7%) [21, 22]. Further, researchers have found that unemployment during young adulthood is positively associated with depression [23–25]. Using results from the 1979–1994 National Longitudinal Survey of Youth, researchers found that being currently unemployed or out of the labor force was positively associated with depression among adults 29–37 [25]. Additionally, using 2010 Behavioral Risk Factor Surveillance System survey data, researchers found that among emerging adults aged 18 to 25, depression was more likely among the unemployed (23.4%) than the employed (8.4%) [24]. Moreover, a systematic review of 20 studies published between 2004 and 2014 showed that job insecurity and unemployment were significantly related with higher depression [23].

#### Unemployment and food consumption

Researchers have found that unemployment status is significantly associated with decreased consumption of fruits and vegetables across age groups [26, 49–51]. For instance, results from the 1990–2009 U.S. Behavioral Risk Factor Surveillance System survey indicate that at the population level, a 1% increase in the unemployment rate in the state in which one resides is correlated with a 3–6% reduction in the consumption of fruits and vegetables among those at the highest risk of being unemployed [26]. Interestingly, this impact is slightly higher in young adulthood than at other ages [26]. Further, an examination of fruit and vegetable consumption among Icelandic adults during the Icelandic economic crisis showed that fruit and vegetable consumption reduced during the crisis at an estimated 5% for vegetables and 10% for fruits [49].

Furthermore, five studies examined the relationship between unemployment and fast food or soda consumption, and there have been some mixed findings [26, 49, 52–54]. According to data from the National Longitudinal Survey of Youth-1979, being unemployed is associated with fewer fast food purchases [53]. Additionally, researchers who examined the impact of the 2008 Icelandic economic crisis on soda and fast food consumption found that being unemployed was associated with decreased consumption of both; for fast food consumption, this relationship was explained almost entirely by higher mortgage debt the crisis caused [49]. Researchers also examined fast food consumption among overweight and obese pregnant women and they found that women who were unemployed ate more fast food than their employed counterparts [52].

## The current study

In this study, we examine the relationship between young women’s consumption of healthy and unhealthy food and depression and explore the moderating effect of their employment status on the relationship. No studies were found that examine unemployment as a moderator between food consumption and depression, regardless of age or gender. Given that young adults tend to be at higher risk of depression compared to those in other life stages [8, 10] and females are more likely to experience depression than males [7–14], it is necessary to pay more attention to young women’s mental health. Even though women’s labor force participation is growing [55], discrimination between males and females still exists in the workplace [56, 57] and unemployment caused by the discrimination may influence levels of depression. Previous studies have addressed food consumption and depression in young adulthood (e.g., [15–18]), but few studies have considered the relationship between both healthy and unhealthy foods and depression in young women and the effect of employment status on the relationship. Moreover, much of the previous research on these relationships was conducted with samples outside the United States, while in this study we examine if these relationships persist in a United States sample. Therefore, the current study considers both healthy and unhealthy food consumption and the moderating effect of employment on the relationship. Research questions in this study are as follows: (1) Does the consumption of healthy and unhealthy foods influence depression among young women?; and (2) Does employment status moderate the relationship between the consumption of healthy and unhealthy foods and depression?

## Methods

### Data and sample

The National Longitudinal Survey of Youth 79 for Children and Young Adults (NLSY79 CY) was used for this study. The U.S. Department of Labor oversaw data collection, and a nation-wide representative sample of individuals participated in the survey. The NLSY79 CY provides information regarding economic factors, food consumption, and demographics. The current study employed the latest wave collected in 2014. Since this study focuses on young women, we limited the sample to females aged from 18 to 35 years. Those who were not interviewed (non-interview) or refused to report their levels of depression were excluded from the study. The final sample included 1524 young women.

### Measures

For healthy and unhealthy foods, the four food types-vegetables, fruits, fast foods, and soft drinks or soda-were classified into two categories because this study focuses on differences between those who ever eat the foods and those who never eat them. This criterion was used in another study examining healthy behaviors [58].

### Depression

The Center for Epidemiologic Studies Depression Scale (CES-D) was used to measure levels of depression. We used the CES-D short form [59, 60]. This measure is comprised of eleven items with a four-point Likert-type scale. Four response options were available from 0 "[I experience this symptom] rarely or none of the time (1 day a week)" to 3 "[I experience this symptom] most or all of the time (5–7 days)." Specific symptoms were as follows: “I did not feel like eating; My appetite was poor;” “I felt I could not shake off the blues, even with help from family or friends;” “I had troubles keeping my mind on what I was doing;” “I felt depressed;” “I felt that everything I did was an effort;” “My sleep was restless;” “I was happy;” “I felt lonely;” “I felt sad;” “I could not get going;” and “I felt life was not worth living.” One item was reverse-coded before analysis. Total scores were computed as the sum of all items. The CES-D scale items were loaded on a single factor with a Cronbach’s alpha of 0.81. The brief 11-item version CES-D was correlated with the full 20-item version CES-D [59]. Higher scores on the depression scale represented higher levels of depression (Mean = 7.54; *SD* = 4.70).

### Healthy foods

#### Vegetables

Respondents stated how frequently they consume vegetables per week. Seven response options were provided and participants chose one of the options. Participants were asked to respond to the question, "How many times do you eat vegetables a week?” The seven response options included: “I do not eat any vegetables; I eat vegetables one to three times per week; four to six times per week; one time per day; two times per day; three times per day; or four or more times per day.” Based on their frequency of eating vegetables, respondents were classified into two groups: Those who eat vegetables at least once a week (coded = 1) and those who never eat vegetables (coded = 0).

#### Fruit

Respondents were asked to report their frequency of fruit consumption per week. They were asked, "How many times do you eat fruits a week?" They chose one of seven responses: “I do not eat any fruits; I eat fruits one to three times per week; four to six times per week; one time per day; two times per day; three times per day; or four or more times per day.” Respondents were classified into two groups: Those who never eat fruit (coded = 0) and those who eat fruit at least once a week (coded = 1).

### Unhealthy foods

#### Fast food

Respondents were asked to report how many times they eat fast food a week. Seven response options were provided and participants chose one. The response options are as follows: “I do not eat any fast food; I eat fast food one to three times per week; four to six times per week; one time per day; two times per day; three times per day; or four or more times per day.” Two groups were created based on their frequency of fast food intake: Those who never eat fast food (coded = 0) and those who eat fast food at least once a week (coded = 1).

#### Soft drinks or soda

Respondents reported their frequency of drinking soft drinks or soda which contain sugar. In this study, soft drinks or soda exclude diet sodas or carbonated water. Respondents answered the following: “In a typical week, how many times do you have a soft drink or soda that contains sugar?” Respondents selected one of seven response options: “I do not typically drink soft drinks containing sugar; I drink soft drinks containing sugar one to three times per week; four to six times per week; one time per day; two times per day; three times per day; or four or more times per day.” Based on their frequency of drinking soft drinks or soda, they were classified into two groups: Those who never drink any soft drinks or soda (coded = 0) and those who drink soft drinks or soda at least once a week (coded = 1).

#### Employment

Respondents reported their employment status via the following question: “Besides your military service, are you currently employed?” Respondents answered either yes or no. Those who responded no were classified as unemployed (coded = 0) while those who answered yes were classified as employed (coded = 1).

#### Control variables

Socio-demographic variables that may also influence depression were controlled for in this study (e.g., [61–65]). Young women’s age, race/ethnicity, marital status, education, and income were included. Race/ethnicity consisted of three groups: Non-Hispanic White, African American, and Hispanic. Marital status was classified into two groups: Married and non-married. Education had two categories: Higher education and non-higher education. Income included wages, salary, commissions, and/or job-related travel before taxes.

### Analysis strategies

**Fig. 1:**
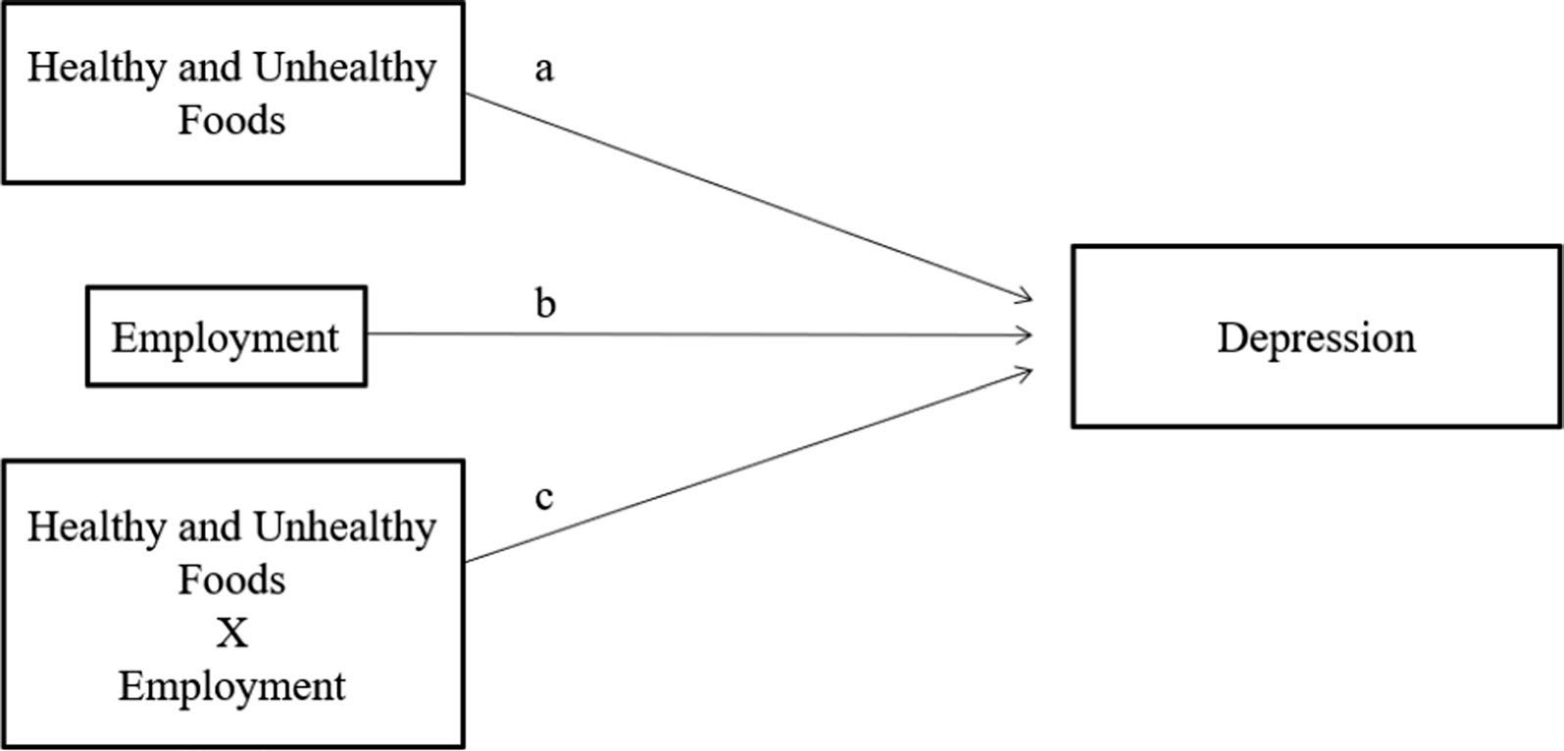
Moderation analysis

Multiple Linear Regression was conducted to examine whether the consumption of healthy and unhealthy foods influences young women’s depression (Research question 1). Model 1 included only healthy and unhealthy foods, and model 2 then considered socio-demographics and the moderator. Based on the results of model 2, we tested if the relationship between the consumption of healthy and unhealthy foods and depression among young women differs by employment status (Research question 2). The Statistical Package for the Social Sciences (SPSS) version 22.0 was used to answer the research questions. Moderation analysis as suggested by [66] and [67] was employed to identify the moderating effect. Three main relationships were identified to test the moderating effects on the relationships between healthy and unhealthy foods and depression among young women. First, we explored the association between healthy and unhealthy foods and depression (a). Second, we examined whether the impact of baseline employment statistically influences depression (b). Lastly, we investigated whether the interaction between healthy and unhealthy foods and employment was significantly associated with depression (c) (Fig. 1). All methods were carried out in accordance with relevant guidelines and regulations provided by the NLSY79 CY.

## Results

As shown in Table 1, 62.7% of young women ate fast foods and 61.0% consumed soft drinks or soda that contains sugar at least once a week. Compared to the prevalence of eating unhealthy foods, the prevalence of consuming fruits and vegetables at least once a week was higher (94.7% for fruits and 96.1% for vegetables, respectively). Further, respondents’ average age was 26.0 years old and about 25% of respondents received any higher education. Additionally, 13% of the young women were married and 83% were employed, while their average income was $13,812.85. Moreover, the racial makeup of the sample was 51.9% non-Hispanic White, 28.2% African American, and 19.9% Hispanic.

**Table 1:** Descriptive statistics for variables included in the study

Variable	Total
	(n = 1524)
Depression	7.54 (4.70)
Unhealthy foods	
Fast foods	62.7%
Soft drink or soda	61.0%
Healthy foods	
Fruits	94.7%
Vegetables	96.1%
Socio-demographics	
Age	26.0 (4.57)
Higher education	24.1%
Marriage	13.1%
Race/ethnicity	
African Americans	28.2%
Hispanics	19.9%
Income	1.38 (1.85)
Employment	83.0%

The real values of income should be multiplied by 10,000

Table 2 showed the relationship between young women’s consumption of healthy and unhealthy foods and depression. Model 1 revealed that fast food consumption was related to higher levels of depression (β = 0.79, *p* < .05) while fruit intake was associated with lower levels of depression (β = − 3.29, *p* < .001). Further, even when socio-demographics were entered into model 2, the effect of fast food and fruit consumption on depression still remained significant (β = 0.76, *p* < .05; β = − 2.98, *p* < .001). Moreover, younger age and being married were negatively related to levels of depression (β = 0.12, *p* < .05; β = − 1.19, *p* < .05), while young women who received any higher education and were employed were also less likely to be depressed (β = − 0.99, *p* < .01; β = − 1.84, *p* < .001).

**Table 2:** Regression results of unstandardized coefficients (standard error) predicting young females' depression

Variables	Depression		
	Model 1	Model 2	Model 3
(Constant)	11.58 (1.16)	10.31 (1.80)	18.41 (2.27)
Unhealthy foods			
Fast foods	.79 (.38)*	.76 (.37)*	− .35 (.85)
Soft drink or soda	.43 (.37)	.01 (.37)	.10 (.36)
Healthy foods			
Fruits	− 3.29 (.83)***	− 2.98 (.81)***	− 11.92 (1.86)***
Vegetables	− 2.00 (1.05)^+^	− 1.53 (1.02)	.05 (1.05)
Socio-demographics			
Age		.12 (.05)*	.09 (.05)^+^
Higher education		− .99 (.38)**	− .98 (.37)**
Marriage		− 1.19 (.48)*	− 1.28 (2.02)**
Race/Ethnicity			
African Americans		.30 (.43)	.36 (.42)
Hispanics		− .80 (.45)^+^	− .69 (.45)
Income		− 1.08 (.00)	− 8.59 (.00)
Employment		− 1.84 (.47)***	− 12.84 (2.02)***
Fast foods*Employment			1.32 (.91)
Fruits*Employment			10.70 (2.01)***

^+^*p* < .10; *p < .05; **p < .01; ***p < .001

**Fig. 2:**
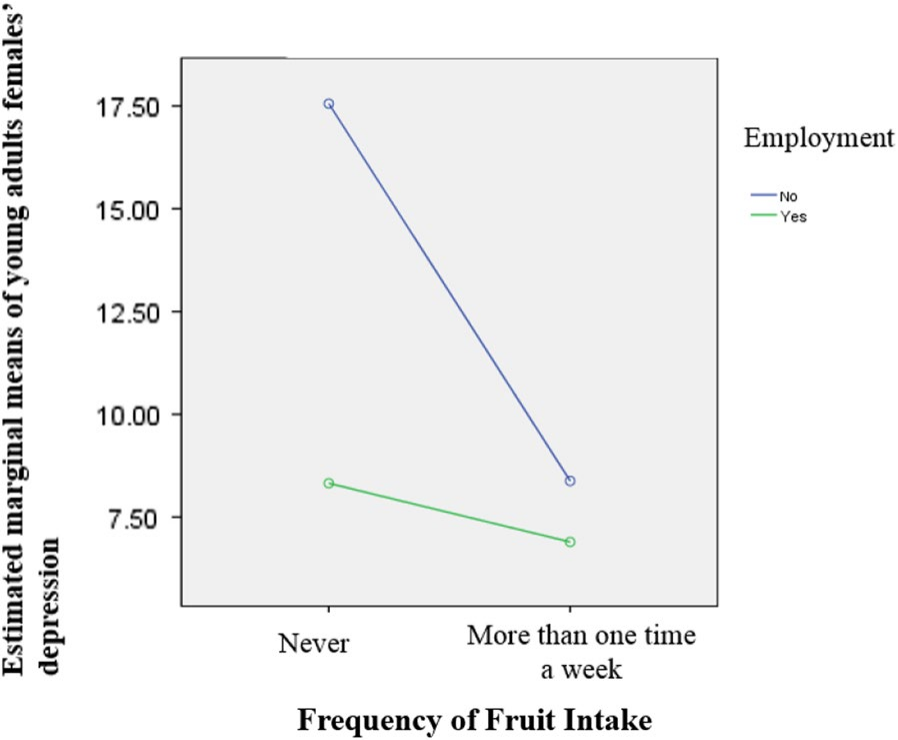
Fruit intake and employment on depression among young female adults

Further, model 3 indicated a significant moderating effect of employment status on the relationship between young women’s fruit consumption and depression (β = 10.70, *p* < .001). Figure 2 shows the moderating effect of employment. Both unemployed and employed young women who ate fruit showed lower levels of depression compared to those who never ate fruit (17.56 vs 8.38 for unemployed women; 8.32 vs 6.90 for employed women). However, the effect of fruit consumption on depression were different depending on employment status. Depression among unemployed young women was greatly influenced by fruit consumption, while depression among employed women was not (9.18 vs 1.42). In other words, depression of unemployed young females was highest if they never eat fruit; however, their level of depression was similar to the level of depression of the employed young females if they ate fruit.

## Discussion

Given that young adults have poor nutrition [1–6, 32, 33] and that young women are at greater risk of depression compared to young men and counterparts in other life stages [7, 8, 10, 11, 14, 40, 41, 43], this study replicated the effect of food consumption on depression among young women. Of all foods included in this study, fast food and fruit consumption were related to depression among young women. In addition, as young women’s labor force participation is increasing, this study considered young women’s employment status, and moderating effects of employment were found: Employment status moderated the relationship between fruit consumption and depression in young women.

In this study, healthy and unhealthy foods were associated with depression in young women. This confirmed previous studies that indicated food consumption was related to mental health (e.g., [15, 16, 18]). Although the research pointed out the importance of food consumption to reduce depression, few studies examined healthy and unhealthy food consumption, particularly among young women. This study confirmed that fast food and fruit consumption are important to reduce levels of depression in young women aged 18 to 35 years old. Given that people with lower incomes and the unemployed are more likely to consume fast food (e.g., [68]) and may not able to purchase fresh fruits, those who often consume fast food and eat fewer fruits may face more economic difficulties, leading them to be at higher risk for depression. Moreover, interestingly, soda and vegetable consumption were not related to depression among young women. Young adults may be more likely to consume soda because they have grown up consuming a variety of such beverages and they may often consume them instead of water. That is, young adults may regard soda as a daily food. In addition, vegetables are an important food for health, but their taste and effects may not be comparable to those of fruits. Therefore, these types of foods may differentially impact people in other stages of life, depending on each generation’s preferences.

Further, the current study revealed the moderating effect of young women’s employment status on the relationship between fruit consumption and depression. As employment is an important factor for depression [23–25] and young women’s labor force participation is increasing [55], this study considered the moderating effect of employment on the relationship between food consumption and depression among young women. A possible reason why employment moderated only the relationship between fruit consumption and depression but not fast food consumption and depression might be related to cost. Generally, fruits are more expensive than fast food, so that employed women may be more likely to buy fruits than unemployed women. That may be why this study revealed a moderating effect of employment on the association between fruit consumptions and depression.

Additionally, both unemployed and employed women showed lower levels of depression if they consumed fruit. In other words, fruit consumption is important for women, regardless of their employment status. However, the inverse effect of fruit consumption on depression was stronger among unemployed women than employed women. Unemployed women reported more severe depression than employed women, which may explain why employed young women’s depression was less influenced by fruit consumption. As unemployed women who consumed fruit showed marked decreases in depression, this suggests the importance of fruit consumption among unemployed young women. Given that there is discrimination between men and women in labor market [56], women who were unemployed due to labor discrimination are often exposed to higher risk of depression compared to unemployed men because they are discriminated by gender. Thus, fruit consumption may be more important for unemployed women who experienced labor discrimination. Furthermore, unemployed women may experience poverty due to interruption of income or low wages in their future jobs as they may be more willing to take a job with low pay in order to get out of unemployment. This negative cycle could lead them to live in low-income areas that make it more difficult to find affordable and healthy foods, in so called “food deserts.” As a result, the opportunity to buy fruits might be very limited among unemployed women and this may result in frustration and hopelessness. Thus, the effect of fruit intake on depression was greater among women who were not employed compared to employed women who can access fruits relatively more easily.

### Limitations

Although this study’s findings contribute to understanding the role of employment in the relationship between food consumption and depression among young women, our results should be interpreted in the context of limitations. First, while we examine the consumption of fast food, soda, fruit and vegetables, additional foods may influence young females’ depression. For instance, snacks and tea consumption or other dining out habits should be considered in future studies. Second, this study focuses on unemployment as a possible moderator; however, as shown in Table 2, educational attainment and marital status may also be a moderator in the relationship between fruit consumption and depression in young women. We recommend that future studies consider other possible moderators to understand the relationship more deeply. Third, responses about food consumption and depression were self-reported. Thus, these responses should be considered within the context of social desirability bias. Fourth, as we mentioned first, this study used a cross-sectional approach. Thus, it is not possible to identify a cause and effect relationship between food consumption and depression among young women. Thus, we recommend that future research employs longitudinal data to make up for this study’s limitation.

### Implications for practice and/or policy

Nutritional programs targeted toward young women should be developed to encourage them to eat healthier foods, particularly fruit, and eat less fast food, perhaps by providing vouchers for local farmer’s markets or other financial assistance to afford healthier food, or no-cost cooking classes in the community to teach simple recipes to address the convenience of eating fast food. Further, given that unemployed women may not have sufficient resources to purchase fruit, programs that are utilized by unemployed women may consider addressing their nutritional needs, perhaps by providing access to nutritious foods or education on the importance of eating healthy foods, especially fruit, for one’s physical and mental health. Organizations that provide mental health or other services to women may also consider having a certified nutrition specialist on staff to address mental health within the context of nutrition.

## Conclusions

Even though previous studies reported the association between food consumption and depression (e.g., [15, 18, 69–71]), little is known about what types of food are effective in reducing young women’s depression, particularly for those who are unemployed. This study’s findings contribute to understanding the importance of young women’s fruit consumption, particularly if they are unemployed.

## Data Availability

The datasets analyzed during the current study are available in the National Longitudinal Surveys repository, [https://www.nlsinfo.org/content/cohorts/nlsy79-children].
